# Overwintering aggregation patterns of European catfish *Silurus glanis*

**DOI:** 10.1186/s40462-023-00373-6

**Published:** 2023-02-07

**Authors:** Samuel Westrelin, Mathieu Moreau, Vincent Fourcassié, Frédéric Santoul

**Affiliations:** 1 INRAE, Aix Marseille Univ, Pôle R&D ECLA, RECOVER, 3275 Route de Cézanne - CS 40061, 13182 Aix-en-Provence Cedex 5, France; 2 Centre de Recherches sur la Cognition Animale, Centre de Biologie Intégrative, Université Paul Sabatier, CNRS, UMR5169, Rue Marianne Grunberg-Manago, 31062 Toulouse, France; 3 Laboratoire Evolution et Diversité Biologique, Université Paul Sabatier, CNRS, ENFA, UMR5174 EDB, 118 Route de Narbonne, 31062 Toulouse, France

**Keywords:** Space use, Fish behavior, Aggregation, *Silurus glanis*

## Abstract

Animal aggregation, particularly in large-bodied species, is both a fascinating and intriguing phenomenon. Here we analyzed the overwintering behavior of the European catfish, *Silurus glanis* Linnaeus, 1758, the largest freshwater fish in Europe. By tracking 47 subadults and adults in a shallow lake in southeastern France, we reported a consistent aggregative behavior across four successive winters. By implementing time series analysis and Cox proportional hazard models, we investigated the dynamics of these aggregations (formation, stability, dislocation), and the factors that govern it, whether external (temperature, time of the day) or specific to the fish (size, key individuals). These aggregations lasted 1.5–2 months and mainly took place in a single small 4 m-deep area whose environmental conditions (temperature, oxygen, substrate) did not differ from other parts of the lake. In some periods during winter, all tagged fish were aggregated, which suggests that a large proportion of the lake population gathered there. Low temperatures (below 9 °C) triggered the formation of aggregations. They became more stable with decreasing temperatures, while individuals more frequently left the aggregation, preferentially at dusk and at night, when temperatures increased. The largest individuals swam more frequently back and forth to the aggregation. Irrespective of their size, some individuals consistently arrived earlier in the aggregation in winter and left later. This predictable seasonal grouping of individuals and, more generally, the knowledge provided by such studies on how species use space have important operational value and are useful for species conservation as well as for species control.

**Supplementary Information:**

The online version contains supplementary material available at 10.1186/s40462-023-00373-6.

## Introduction

Aggregations of individuals are widespread in the animal kingdom, spanning a large range of sizes and time durations [45]. Though they have been observed for a long time, they still remain striking and intriguing for biologists [33]. Aggregations are particularly common among fishes, both marine and freshwater, most of which form cohesive social groups at some stage of their life [48]. They have been shown to bring numerous benefits, including protection from predators [26, 35], increased probability of encountering a mate [12, 16, 21], increased foraging efficiency [17, 25], reduction of energy expenditure [24, 38] and centralized information [42]. These benefits are assumed to outweigh the costs incurred by aggregations, such as within-group competition for resources [70] and exposure to parasites [63, 67]. On an evolutionary time scale, aggregation behavior is thus expected to increase individual fitness [27]. Aggregations in fish range from relatively small shoals, usually in freshwater streams and lakes (e.g. [15, 22, 31, 68], to structured schools of up to hundreds of thousands of individuals in marine systems (e.g. [69]. In most fish species, aggregations are stable over time [57], but for others they can be transitory, for example when fish are attracted by aggregating devices (e.g. [41]) or when they spawn (e.g. [12, 16, 46]).

In addition to spawning, in some species, temporary aggregations have been described for overwintering, a phase in which fish individuals are exposed to numerous stressors, e.g. starvation, thermal stress, but this has rarely been studied [59]. In freshwater systems, these wintering aggregations have long been described in common carp *Cyprinus carpio* [3, 29, 46, 48, 65] and more recently in another large and long-lived species, the lake sturgeon *Acipenser fulvescens*, the largest freshwater fish in North America [59]. Winter aggregations also occur in centrarchid fishes during the light phase of the day [58]. However, it is still an understudied aspect of their ecology. As regards to European catfish *Silurus glanis*, the largest European freshwater fish, only a single study has reported the existence of repeated aggregations involving 15–44 adults [5]. These aggregations occurred throughout the year at the same place in a large river [4]. Brevé et al. [6] also observed an aggregation of catfish adults in a river section underneath boats, but just once. Yet, in a small shallow eutrophic lake, Vanovac et al. [65] found that while common carp were aggregating during winter, a stocked population of European catfish was not. European catfish are reported to be solitary foragers [11], that preferentially feed at night. They expend more energy when in contact with conspecifics in preferred areas of habitat, probably to monopolize resources [52], at least in their native range. Aggregations in European catfish warrant further work [14].

While the ultimate causes of aggregations have been extensively studied and, in some cases, are well established, the proximate mechanisms that underlie the formation and dislocation of aggregations are less understood [62]. The transitory nature of winter aggregations provides an opportunity to study these mechanisms. Moreover, most studies on animal aggregations have dealt with collective coordinated behaviors that emerge from interactions between individuals that are considered equivalent [57]. Yet, there is a growing body of evidence that individual variability can play an important role in these aggregations [30] and recent advances in high resolution tracking of individuals now provide tools to investigate its role in aggregation dynamics [43].

In this paper, we present the results of an experiment in which 47 subadults and adults of the European catfish were tracked by acoustic telemetry for 4 years in a shallow eutrophic lake located in southeastern France. We focus on their overwintering behavior by analyzing their movements over four successive winters showing contrasted temperatures; we found that every winter, catfish exhibited an aggregative behavior. We investigated the dynamics of these aggregations (formation, stability, dislocation) as well as the factors that could govern it, whether related to external conditions (temperature, time of the day) or to the characteristics of the fish (size, key individuals).

## Material and methods

### Study site

**Fig. 1:**
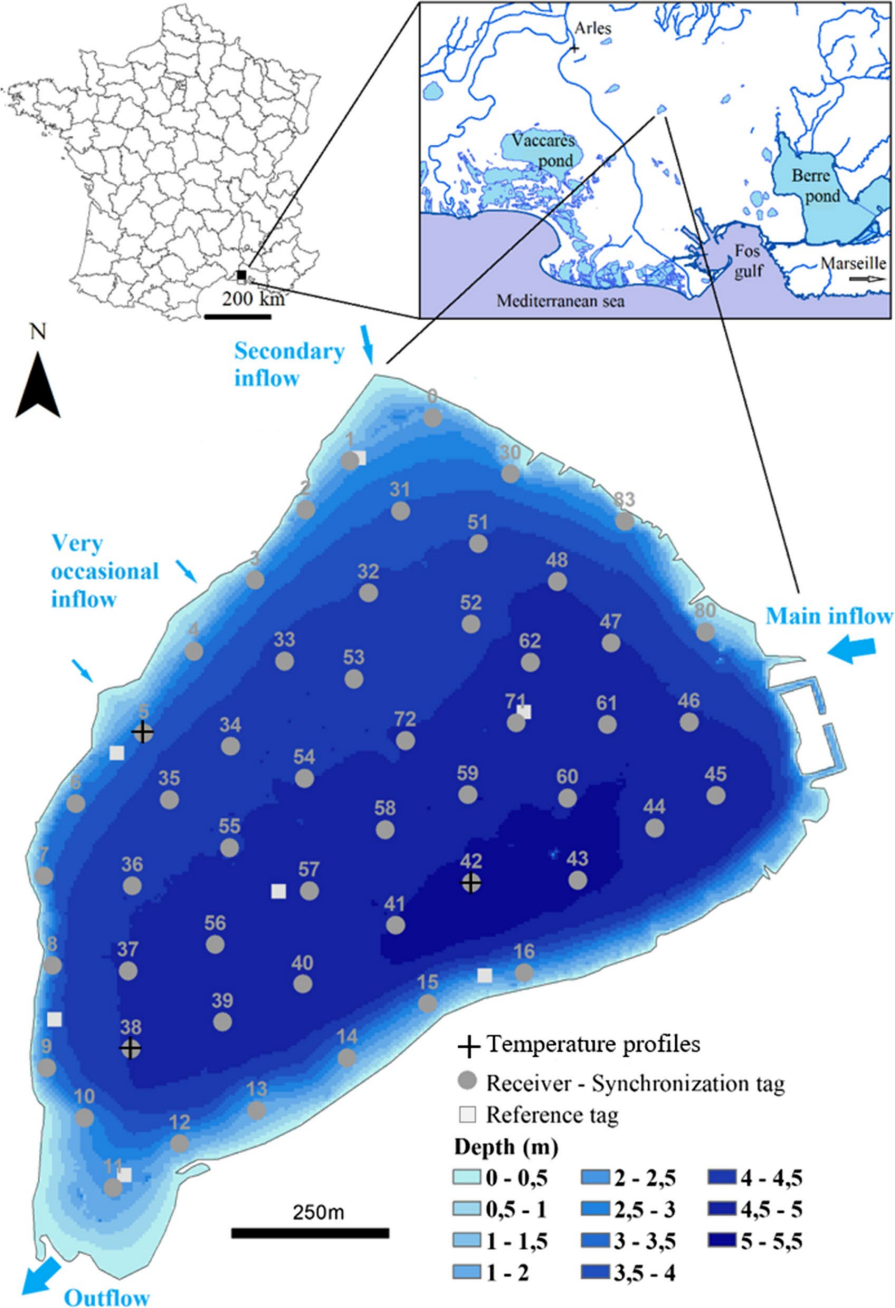
Bathymetric map of “Etang des Aulnes” and experimental setup. The bathymetry was calculated at the water level of 11.14 m above sea level. Acoustic receivers and their associated synchronizing tags are represented by grey dots. Reference tags are symbolized by pale grey squares. Hourly temperature profiles are indicated by crosses; location 42 is the deepest in the lake

“Etang des Aulnes” is a 104-ha shallow natural lake of 3.8 m mean depth and 6 m maximum depth, located in South-Eastern France in a protected natural area (Fig. 1). The fish assemblage in the lake, determined by fyke nets, fishing traps and electro fishing in October 2017, 2018 and 2019 was composed of 16 species. The most dominant species were common bream (*Abramis brama*, relative abundance 65%), European perch (*Perca fluviatilis*, 13%), pumpkinseed (*Lepomis gibbosus*, 8%), tench (*Tinca tinca*, 4%), pikeperch (*Sander lucioperca*, 4%), European catfish (*Silurus glanis*, 3%) and Northern pike (*Esox lucius*, 2%). In addition, two crayfishes were present: *Procambarus clarkii* and *Faxonius limosus*. Fishing is allowed but only during daytime from the eastern bank of the lake and no other activity is authorized.

### Physical and chemical lake conditions

The “Etang des Aulnes” is eutrophic (see [71] for chemical element concentrations). Hourly temperature profiles were recorded with HOBO data loggers U22 (0.2 °C accuracy) at the deepest point in the lake (location 42 on Fig. 1) and at several other locations, among which points 5 and 38; they are presented later. During the winter period, temperatures are mainly homogenized in the water column; the characteristics of each winter are given in Table 1. At the deepest point in the lake, hourly profiles of dissolved oxygen concentration were also recorded with HOBO data loggers U26 (0.2 mg/L accuracy).

**Table 1:** Temperature and catfish characteristics in each winter from 2017 to 2020

	Winter 2017	Winter 2018	Winter 2019	Winter 2020
Period	12/02/2017 17:15 01/21/2018 11:15	12/03/2018 06:15 03/06/2019 20:30	11/10/2019 06:00 03/02/2020 05:30	11/22/2020 14:00 02/06/2021 19:00
Daily temperature (°C)	6.5 *(1.5)* [4.7; 8.9]	6.8 *(2.3)* [3.3; 11.4]	9.5 *(1.3)* [6.9; 13.9]	7.0 *(2.0)* [3.2; 11.0]
All individuals				
n	10	42	42	42
Total length (mm)	893 *(187)* [727; 1465]	1028 *(309)* [727; 2150]	1056 *(326)* [727; 2150]	1064 *(313)* [727; 2150]
Small < 850 mm				
n	5	9	9	8
Total length (mm)	791 *(33)* [727; 839]	812 *(34)* [727; 847]	812 *(34)* [727; 847]	812 *(36)* [727; 847]
Medium [850; 1100[ mm				
n	4	26	24	23
Total length (mm)	876 *(15)* [855; 902]	942 *(59)* [855; 1060]	944 *(59)* [855; 1060]	945 *(59)* [855; 1060]
Large ≥ 1100 mm				
n	1	7	9	11
Total length (mm)	1465	1623 *(335)* [1100; 2150]	1600 *(311)* [1100; 2150]	1495 *(322)* [1100; 2150]

The daily temperature (mean, *(sd)* and [range], in °C) at 3 m above the bottom of the lake at its deepest point (point 42 on Fig. 1), the number of tracked catfish (n) and their total length (mean, *(sd)* and [range], in mm) for all pooled individuals and for each class size are given for the extended aggregation period analyzed in each winter

Values in italics and in parenthesis correspond to the standard deviation of the parameter

### Fish tagging

Specifically, a total of 47 catfish, subadults and adults, were caught by fyke nets, angling or electrofishing over four sampling campaigns: 10, 32, 2 and 3 individuals, respectively in October 2017, 2018, 2019 and 2020. Once caught, fish was anesthetized by immersion for 5–6 min in a benzocaine solution (80 mg/L). A 15–20 mm-long incision was made on the ventral side to insert the tag and was closed using 2–3 simple surgical sutures (3–0 Polydioxanon resorbable monofilament). The surgery itself took 5–6 min while the fish was in another solution of benzocaine (40 mg/L). Fish recovered in 10 min and were released after 3–6 h. The surgical procedure followed for fish tagging is detailed in Westrelin et al. [71]. At the end of 2018 and 2019 winters, respectively 2 and 3 tags were stationary and, consequently, the corresponding individuals were discarded from the analyses as they were assumed to be dead or to have expelled their tag. Vemco V13-1L acoustic transmitters (length: 30.5 mm, weight: 9.2 g in the air, mean battery life: 1825 days, mean burst interval: 180 s—range 120–240 s—for the 12 used in 2017 and 320 s—range 260–80 s—in 2018) were used. The transmitter weight in the air did not exceed 2% of the fish body weight [55, 72]. The characteristics of the tracked fish are given in Table 1.

### Fish tracking

Fifty-two underwater omnidirectional Vemco acoustic receivers (20 VR2W 69 kHz and 32 VR2Tx 69 kHz) with their associated synchronization tag (additional V16-1L transmitter for VR2W and built-in V16-like transmitter for VR2Tx, 500–700 s), used to correct for receiver internal clock drift, were anchored to the bottom of the lake in October 2017 (Fig. 1). Seven reference tags (V13-1L, 840–960 s) were added to detect anomalies in the tracking system. On average, neighboring receivers were positioned 155 m from each other (range, 100–209 m), at a depth of 3.9 m (range, 1.5–6 m), 0.5 m above the bottom of the lake. Receivers were removed roughly every 6 months to download fish detections. From these detections, fish 2D positions were calculated with the Vemco Positioning System (VPS) [54]. The horizontal position error, a dimensionless parameter calculated by the VPS for each position, gives information on the quality of the position estimate, and was used to filter the data set [20]. Here, we retained only positions with horizontal position error below 100; this limit represented a good compromise between the mean position error (7.4 m, calculated on reference tags) and the percentage of positions kept (87%).

### Space use metrics

Fish were continuously tracked from October 2017 onward. As our study focuses on the winter period, only data recorded from the 15th of October to the 15th of March of the next year were analyzed. To obtain synchronized individual tracks, individual raw positions were interpolated using the R package *trajr* [40] for each quarter hour between the first and the last position recorded. In order to visualize catfish space use in winter, all tracks were then plotted on the lake map to create videos of the catfish displacements (Additional file 1). The videos clearly show an aggregation localized in the western part of the lake.

To identify the aggregation zone over the 5-month periods, the home ranges of the pooled fish were estimated with an Epanechnikov kernel as the utilization distribution with probability levels 95%, 50% [74], and the level corresponding to the highest percentage delineating the aggregation zone identified on the videos. The home ranges were estimated for each phase of the daily cycle, i.e. dawn, day, dusk and night, defined at an hourly resolution. Dawn was defined as the period including the hour preceding the sunrise hour, the sunrise hour itself and the following hour. Dusk was defined as the period including the hour preceding the sunset hour, the sunset hour itself and the following hour. Dawn and dusk lasted 3 h each. Daytime was the period following dawn and preceding dusk and night was the period following dusk and preceding dawn. These spatial analyses were conducted using the R package *adehabitatHR* [9]. To quantify the degree of aggregation over time, the mean distance between individuals was calculated each quarter hour using the R package *spatstat* [2] and the number of fish in the aggregation zone was counted.

### Statistical analyses

To isolate the aggregation period, we applied an algorithm to detect possible breakpoints corresponding to structural changes in the 5-month time series of the mean distance between individuals (*strucchange* R package, [75, 76]. We considered that for each of the four winters, the periods with the lowest mean distances between individuals corresponded to the time at which aggregation occurred; these periods were confirmed by watching the videos. To investigate the formation and dislocation of the aggregation, these periods were extended to before, when no individual had joined the aggregation zone yet, and after, until all individuals had left it. These periods will hereafter be referred to as the extended aggregation periods.

To investigate whether certain individuals consistently joined the aggregation earlier than others at the beginning of the aggregation period, we performed a comparison of the ranks at which individuals first joined the aggregation over successive winters using a Friedman test. To highlight whether fish size influenced the timing of aggregation, we then compared the mean rank of joining the aggregation of the different size classes with a Kruskall-Wallis test. Fish size was defined from the total body length measured during tagging and was categorized into three classes: “Small”, “Large” and “Medium”, corresponding to total length < 850 mm, ≥ 1100 mm and in-between, respectively (Table 1). Fish were only measured during tagging as very few were recaptured; we hypothesized they did not switch to other size-classes during the study. The same analysis was performed at the end of the aggregation period, when the aggregation disbanded, by considering the rank at which individuals permanently left the aggregation. To quantify a possible link between the mean rank of arrival in and the mean rank of departure from the aggregation, a Spearman correlation coefficient was calculated. These analyses were only performed over the last three winters, when a common significant pool of individuals was present (38 individuals throughout these 3 winters).

To investigate the effect of temperature, time of day and fish size on the stability of winter aggregations, we used two multivariate mixed effects Cox proportional hazard models (coxme R package [60]). The first model was used to assess the effect of the covariates on the rate of temporarily leaving the aggregation for an excursion. While the second model was used to assess the effect of the covariates on the rate of returning to the aggregation after an excursion. The two models can be formulated as follows:

- Survival(Start, Stop, Event) ~ TEMPERATURE + FISH SIZE + TIME OF DAY + (1|Fish identity)

where TEMPERATURE is the temperature at 3 m above the bottom of the lake at its deepest point, FISH SIZE is the fish class size (3 levels: Small, Medium, Large) and TIME OF DAY is the day period (4 levels: Dawn, Day, Dusk, Night).

In the first model, Event corresponds to the “leave the aggregation” behavior; for each Event, the time-to-event ranges from the time at which a given individual joined the aggregation (Start) to when it left it (Stop). In the second model, the Event corresponds to “join the aggregation”; for each Event, the time-to-event goes from the time at which a given individual left the aggregation (Start) to the time at which it joined it (Stop).

Fish identity was considered as a random effect to account for individual variability and repeated measurements made on the same individuals. The Cox models were run on the whole dataset which included the four pooled winters. Since two of the fixed effects (temperature and time of day) varied with time, the dataset was rearranged so that each quarter hour observation of a given individual fish was treated as a separate observation, i.e. containing a Start and Stop time and the corresponding Event type. The effects of significant categorical covariates were further analyzed by Tukey comparisons of pairwise estimated marginal means of their different levels (*emmeans* R package [36]).

The survival functions, which represent the probabilities of time-to-event over time, were estimated using the Kaplan–Meier method [51] and plotted for the different covariates (*survival* R package, [61]. For the temperature effect, the following classes were used to plot the survival curves: [3; 5[, [5; 7[, [7; 9[ and [9; 14] °C; the range of the warmest class is larger to avoid a small sample size as temperatures above 11 °C rarely occurred in winter.

All statistical analyses and graphics were made using R version 3.6.3 [49].

## Results

### Aggregation characteristics

The aggregation period lasted 41.9, 67.4, 55.2 and 59.7 days and the extended aggregation 49.8, 93.6, 113.0 and 76.2 days, respectively in 2017, 2018, 2019 and 2020 (Table 1) (Additional file 2 details the breakpoints analysis). The difference between these two periods corresponds to the time for the formation and dislocation of the aggregation, i.e., 7.8, 26.2, 57.8 and 16.5 days, respectively in 2017, 2018, 2019 and 2020.

**Fig. 2:**
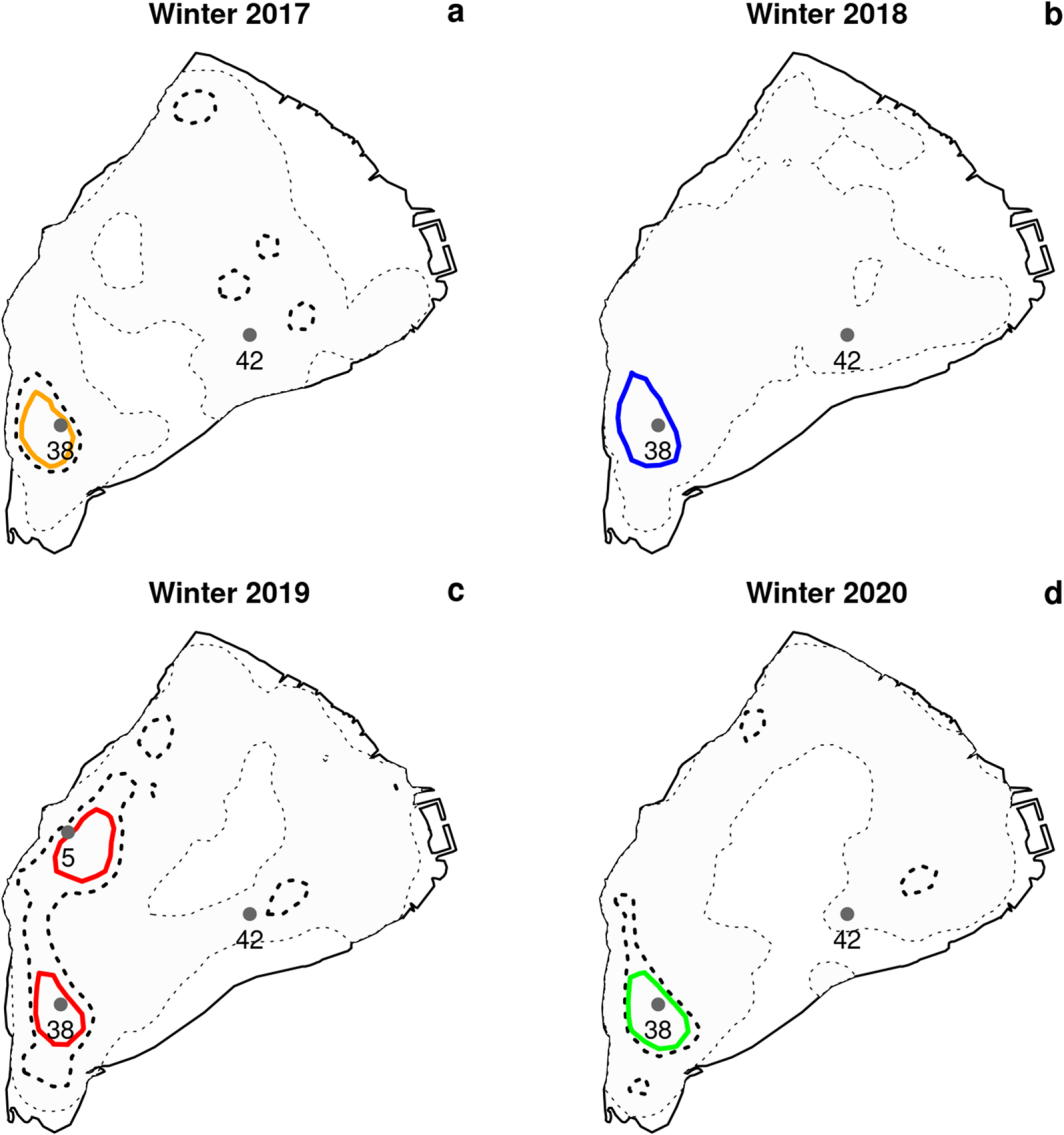
Catfish’s home ranges over 15 october–15 March in winters 2017 to 2020. Home range 95% is filled in pale grey and delineated with a thin dotted line; home range 50% is delineated with a bold dotted line. The contour of the aggregation zone is colored in orange, blue, red and green, respectively in 2017 (**a**), 2018 (**b**), 2019 (**c**) and 2020 (**d**). The aggregation zone corresponds to the utilization distribution with probability level of 35% (21 352 m^2^ area), 50% (32 141 m^2^ area), 30% (44,050 m^2^ area) and 40% (26,035 m^2^ area), respectively in 2017, 2018, 2019 and 2020. The aggregation zone exactly matches with the home range 50% in 2018. The total area of the lake is 1,036,888 m^2^. The grey dots symbolize the locations 5, 38 and 42 where hourly temperature was measured. The points 5 and 38 are close or inside the aggregation zone and the point 42 is the deepest point of the lake which stands as a reference point

**Fig. 3:**
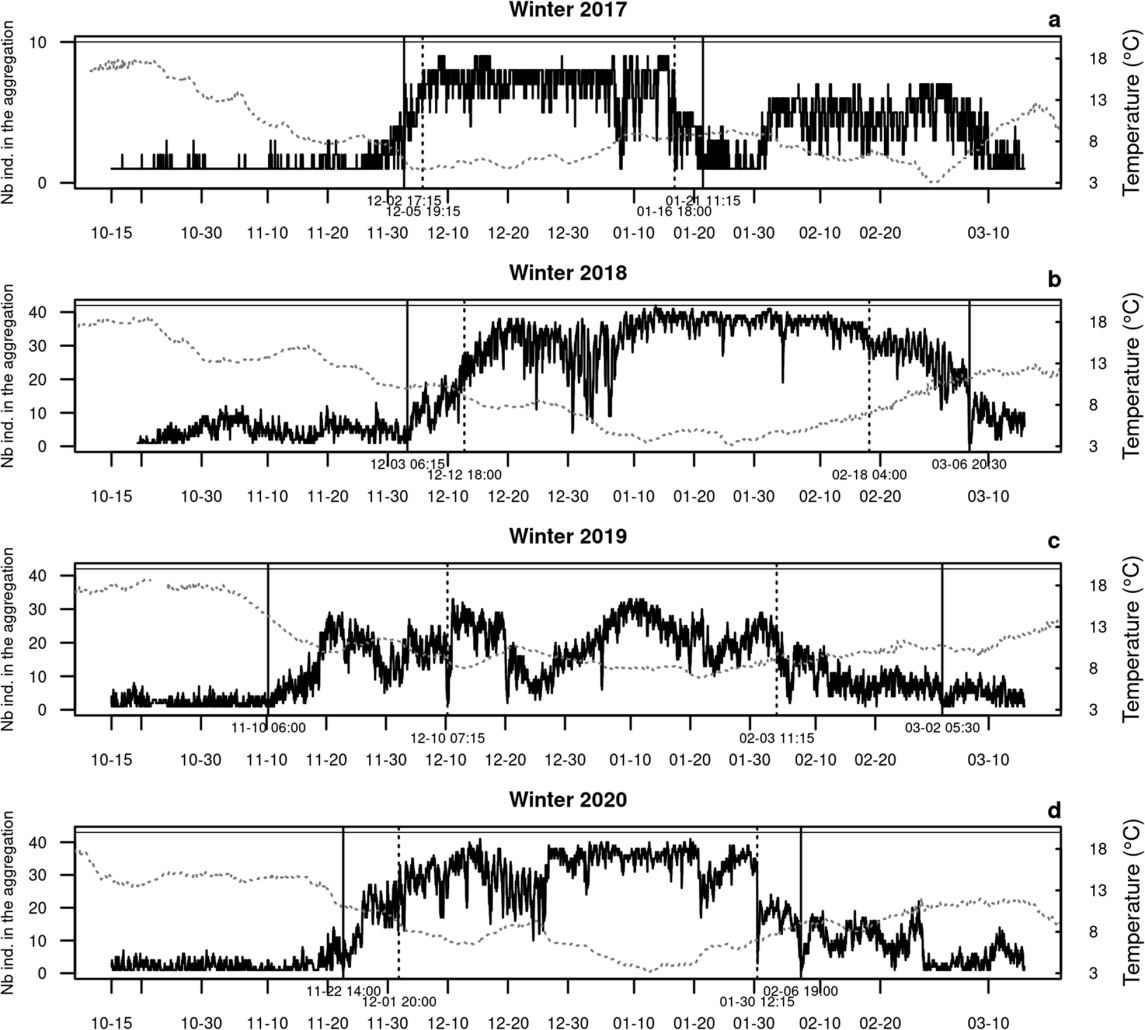
Time series of the number of individuals in the aggregation zone in winters 2017–2020. The dates of aggregation given by the breakpoint detection algorithm are represented by vertical dotted lines. The period extended to the formation and dislocation of the aggregation is delimited by vertical solid lines. On the right y-axis, the temperature at the deepest point in the lake (3 m above the bottom) is plotted in dotted line.The horizontal gray solid line at the top of each panel corresponds to the total number of catfish tracked in the corresponding winter

**Fig. 4:**
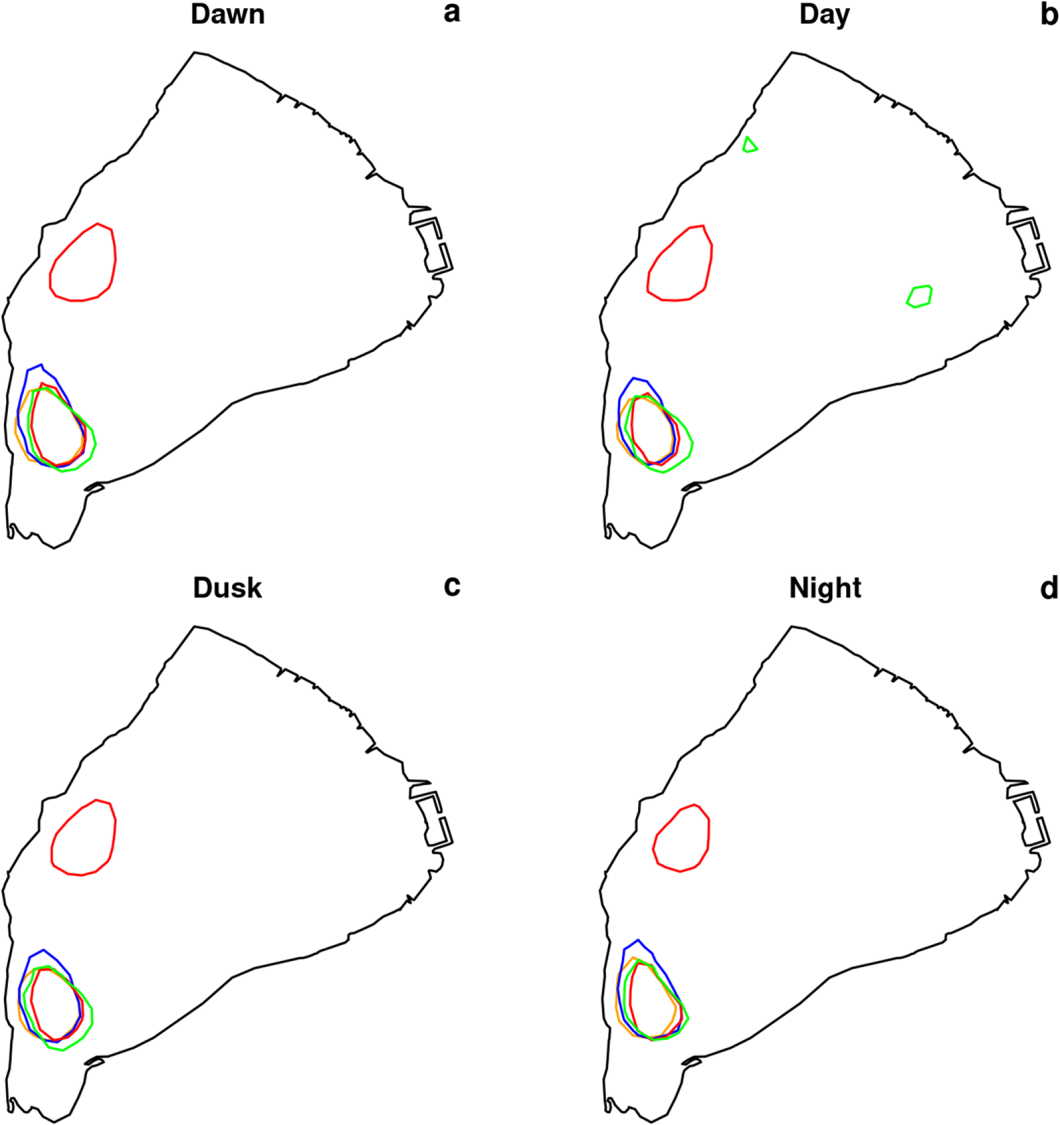
Catfish’s aggregation zone in winters 2017–2020 according to the period of the day. The contour of the aggregation zone is colored in orange, blue, red and green, respectively in 2017, 2018, 2019 and 2020. The plotted home ranges correspond to the utilization distribution of Fig. 2, i.e., with probability level of 35%, 50%, 30% and 40%, respectively in 2017, 2018, 2019 and 2020. Dawn, day, dusk and night are respectively plotted in **a**–**d**

Catfish exhibited strong fidelity to the same aggregation zone across the four winters, although they were more scattered in the milder 2019 winter (Fig. 2, Table 1). The aggregation area ranged between 2.1 and 3.2 ha, if we exclude 2019 when the aggregation was not as dense as in other winters (Additional file 1) and was split into two main zones yielding a total area of 4.4 ha. However, one of the two zones was the same as in the other winters and extended over 2.0 ha (Fig. 2). The aggregation zones corresponded to a probability of utilization of 35%, 50%, 30% and 40% over the 15 October–15 March period, respectively in 2017, 2018, 2019 and 2020, which means that fish spent 30–50% of their time in this zone over this 5-month period. If this spatial analysis is restricted to the extended aggregation period, the probability of utilization rises to 60%, 70%, 40% and 70%, respectively in 2017, 2018, 2019 and 2020. In the 2017, 2018 and 2020 winters, during the identified aggregation period, most individuals were indeed located within the aggregation zone (on average 68.0, 72.4 and 68.9% over the extended aggregation period in 2017, 2018 and 2020 respectively; Fig. 3a, b, d), but aggregation was weaker in 2019 (in average 38.7%, Fig. 3c). The identified aggregation periods corresponded to the time at which the coldest temperatures were recorded (Fig. 3 and Table 1). The aggregation zones were very stable across the different times of day (Fig. 4).

### Environmental conditions

The main aggregation zone, which could be identified throughout the four winters, was on average 4.3 m deep (range [2.8; 4.8] m) and at a distance of 107.2 m from the bank (range [42.8; 196.6] m). The secondary aggregation zone, which appeared only in winter 2019, was on average 4.2 m deep (range [3.7; 4.6] m) and at a distance of 134.1 m from the bank (range [62.5; 215] m). The mean daily temperature differences between the main aggregation zone and the deepest point in the lake, which stands as a reference point, ranged between [− 0.2; 0.4] °C throughout the extended aggregation period and had a 0.0 °C mean over this period (Fig. 2, Additional file 3). The mean daily differences of temperature between a location close to the secondary aggregation zone and the deepest point in the lake ranged between [− 0.4; 0.5] °C throughout the extended aggregation period and had a − 0.1 °C mean over this period (Fig. 2, Additional file 4).

Over the extended aggregation period, at the deepest point in the lake, mean daily oxygen concentrations ranged between [9.8; 14.2], [6.1; 15.1], [9.4; 12.9] and [8.2; 13.7] mg/L, corresponding to saturation rates that ranged between [83.5; 113.9], [48.3; 134.8], [81.3; 112.8] and [70.2; 105.4] %, respectively in 2017, 2018, 2019 and 2020.

### Aggregation dynamics

**Fig. 5:**
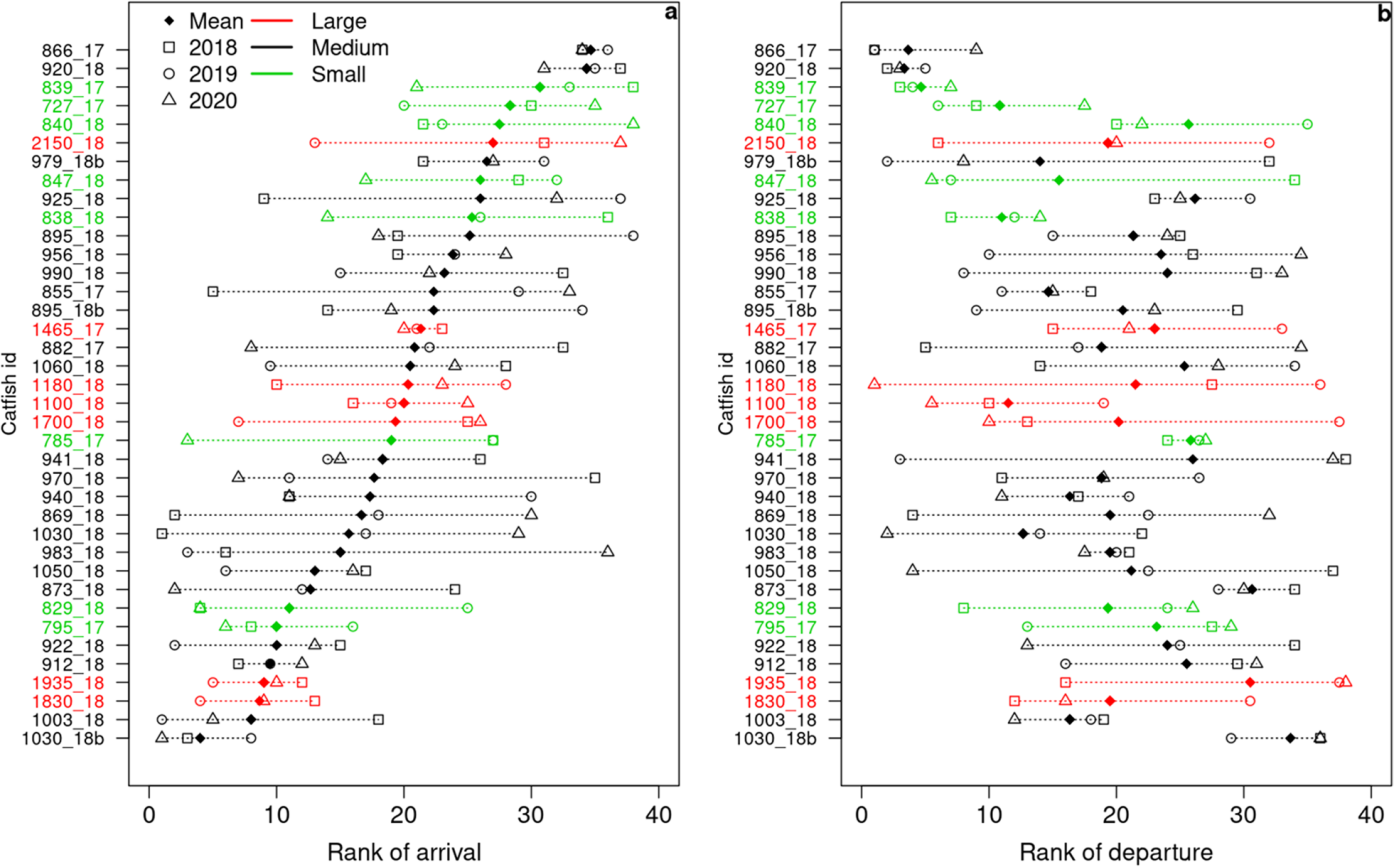
Rank of arrival in and departure from the aggregation of catfish. The 38 individuals present in the 2018, 2019 and 2020 winters are here considered. Individuals are labeled on the y-axis with the following convention: the first part of the label corresponds to the fish total length (in mm) and the two last digits to the year it was tagged; when two fish of the same length were tagged the same year, a “b” has been added at the end of the label of the heavier fish. In a, individuals are ordered by increasing mean rank of arrival; in b, the same order has been kept. Different colors are used for fish of different sizes (green, black and red for small, medium and large, respectively) and different symbols for the three winters (square, circle, triangle and filled losange for 2018, 2019, 2020 winters and the mean rank, respectively). A dotted line joins both extreme ranks among winters for each individual

There was no significant consistency in the rank of first arrival of individuals in the aggregation between the three last studied winters (Friedman test: Chi2 = 51.56, *df* = 37, *p* = 0.056). However, the rank of first arrivals was comparatively consistent for the individual with the lowest mean rank (mean rank 4; range [1; 8] for individual 1030_18b) and for the two individuals with the highest mean ranks (mean rank 34.3 and 34.7; range [31; 37] and [34; 36], respectively for individuals 920_18 and 866_17) (Fig. 5a). Moreover, the rank of first arrival did not depend on fish size (Kruskall-Wallis test: Chi2 = 2.13, *df* = 2, *p* = 0.345), although the mean rank of arrival in the aggregation of 5 out of the 8 small fish was higher than that of 70% of all fish considered in the analysis (n = 38). Neither was there any consistency in the rank of last departure of the fish from the aggregation (Friedman test: Chi2 = 45.10, *df* = 37, *p* = 0.169), nor any size effect (Kruskall-Wallis test: Chi2 = 0.97, *df* = 2, *p* = 0.614). Nevertheless, there was much less variability in the rank of last departure of the three individuals with the lowest mean ranks (mean rank 3.33, 3.67 and 4.47; range [2; 5], [1; 9] and [3; 7], respectively for individuals 920_18, 866_17 and 839_17) and of the two individuals with the highest mean ranks (mean rank 30.7 and 33.7; range [28; 34] and [29; 36], respectively for individuals 873_18 and 1030_18b) (Fig. 5b). The correlation between the mean rank of arrival and mean rank of departure was significantly negative (Spearman rho = − 0.43, *p* = 0.007) meaning that, on average, the first individuals to arrive in the aggregation were the last to leave and vice versa.

**Fig. 6:**
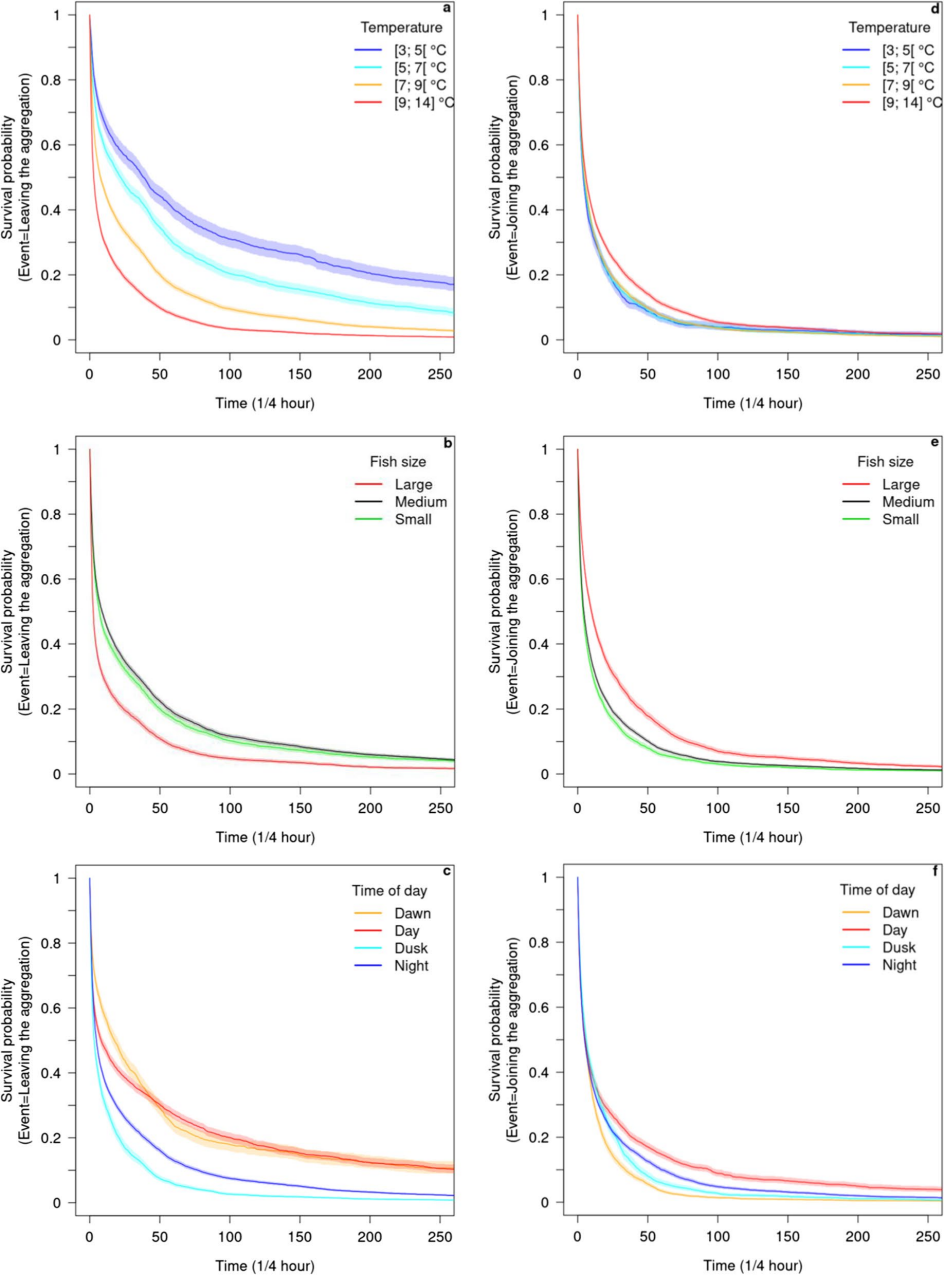
Survival probabilities corresponding to the events “leaving the aggregation” and “joining the aggregation”. The survival probabilities have been estimated by the Kaplan–Meier method for the events “leaving the aggregation” (**a**–**c**) and “joining the aggregation” (**d**–**f**) during the period of winter aggregations for different water temperatures (**a**, **d**), different fish sizes (**b**, **e**) and the different times of day (**c**, **f**). These curves, which represent the survival function as a function of time, show the probability that the event of interest has not yet occurred by this time point. For example, in a, the probability for an individual of not leaving the aggregation after time = 100 (25 h), in other words, the probability of staying in the aggregation after 25 h spent inside, is 0.03 at [9; 14[ °C, 0.10 at [7; 9[ °C, 0.20 at [5; 7[ °C and 0.31 at [3; 5[ °C. The curves have been computed on the four studied winters together. The shading around the curves represents the 95% confidence interval of the mean survival curve. It is sometimes barely discernible because it is very narrow

There was a significant effect of temperature, fish size and time of day on the probability of leaving the aggregation for an excursion (Table 2a, b) and on the probability of coming back to the aggregation after an excursion (Table 3a, b). The probability of leaving the aggregation was significantly higher with increasing water temperature (Fig. 6a, Table 2c), meaning that, on average, fish stayed longer in the aggregation when the water temperature remained low. This probability was similar between small and medium fish but increased for large fish (Fig. 6b, Table 2c, d). Small and medium fish therefore made longer stays in the aggregation than large fish. Moreover, the probability of leaving the aggregation did not vary between dawn and day (Fig. 6c, Table 2d) but increased between day and dusk (Table 2c). It decreased between dusk and night and between night and dawn (Fig. 6c, Table 2c), which means that fish left the aggregation mostly at dusk and at night, but even more often at dusk.

**Table 2:** Results of the Cox model corresponding to the event “Leaving the aggregation”

	Log-likelihood	Chi2	*df*	*p* value
*a*				
NULL	− 139,012			
Temperature	− 137,329	3365.39	1	< 0.001
Fish size	− 137,322	14.64	2	< 0.001
Time of day	− 136,845	954.25	3	< 0.001
*b*				
Model without random effects: Log-likelihood = − 137,257; Concordance = 0.641; Wald statistic = 3421 (*df* = 6, *p* < 0.001) Comparison between model with and without random effects (deviance analysis): Chi2 = 825.04 *df* = 1 *p* < 0.001				

	Coef	Exp(coef)	z	*p*
*c*				
Temperature	0.21	1.23 [1.22; 1.24]	46.70	< 0.001
Large/small	0.37	1.44 [1.11; 1.87]	2.77	0.006
Large/medium	0.44	1.55 [1.26; 1.90]	4.14	< 0.001
Dusk/day	0.68	1.98 [1.88; 2.10]	25.07	< 0.001
Night/dusk	− 0.21	0.81 [0.78; 0.85]	− 9.28	< 0.001
Dawn/night	− 0.45	0.64 [0.61; 0.68]	− 16.24	< 0.001

Contrast	Ratio	*df*	z ratio	*p*
*d*				
Small/medium	1.07	Inf	0.61	0.817
Small/large	0.69	Inf	− 2.77	0.016
Medium/large	0.65	Inf	− 4.14	< 0.001
Dawn/day	1.03	Inf	0.89	0.811
Dawn/dusk	0.52	Inf	− 20.35	< 0.001
Dawn/night	0.64	Inf	− 16.24	< 0.001
Day/dusk	0.50	Inf	− 25.07	< 0.001
Day/night	1.23	Inf	− 21.81	< 0.001
Dusk/night	1.23	Inf	9.28	< 0.001

The model equation is the following: Survival(Start, Stop, “Leaving the aggregation”) ~ TEMPERATURE + FISH SIZE + TIME OF DAY + (1|Fish identity). Part a gives the statistics and associated *p* value of each covariate. Part b compares this model with the same model without random effects and gives the goodness-of-fit of this latter model which is not accessible for the mixed model; the Wald test tests the null hypothesis that the coefficients of the covariates are null; the Concordance should be greater than 0.5 for the model to be informative. Part c shows the covariate coefficients of the mixed Cox model; the exponentiated coefficients are multiplicative effects on the hazard: for continuous covariates, as Temperature, exp(coef) = 1.23 means that when temperature rises by 1 °C, the probability to leave the aggregation increases by 23%. For categorical covariates, for example the coefficient of large fish in reference to small fish, exp(coef) = 1.44 means that the probability for large fish to leave the aggregation is 44% higher than that of small fish. Coefficients are shown only for significant contrasts in part d and, for time of day, only between consecutive classes in the diel cycle (Day/Dawn, Dusk/Day, Night/Dusk and Dawn/Night)

**Table 3:** Results of the Cox model corresponding to the event “Joining the aggregation”

	Log-likelihood	Chi2	*df*	*p* value
*a*				
NULL	− 137,700			
Temperature	− 137,256	889.09	1	< 0.001
Fish size	− 137,248	15.56	2	< 0.001
Time of day	− 137,159	178.78	3	< 0.001
*b*				
Model without random effects: Log-likelihood = − 137,385; Concordance = 0.557; Wald statistic = 619.5 (*df* = 6, *p* < 0.001) Comparison between model with and without random effects (deviance analysis): Chi2 = 452.3 *df* = 1 *p* < 0.001				

	Coef	Exp(coef)	z	*p*
*c*				
Temperature	− 0.06	0.95 [0.94; 0.95]	− 12.94	< 0.001
Large/small	− 0.40	0.67 [0.54; 0.83]	− 3.72	< 0.001
Large/medium	− 0.33	0.72 [0.60; 0.85]	− 3.77	< 0.001
Day/dawn	− 0.37	0.69 [0.65; 0.73]	− 13.27	< 0.001
Dusk/day	0.21	1.23 [1.16; 1.31]	7.08	< 0.001
Night/dusk	− 0.05	0.95 [0.90; 1.00]	− 2.15	< 0.001
Dawn/night	0.22	1.24 [1.18; 1.30]	9.19	< 0.001

Contrast	Ratio	*df*	z ratio	*p*
*d*				
Small/medium	1.08	Inf	0.81	0.700
Small/large	1.50	Inf	3.72	< 0.001
Medium/large	1.39	Inf	3.77	< 0.001
Dawn/day	1.45	Inf	13.27	< 0.001
Dawn/dusk	1.18	Inf	5.21	< 0.001
Dawn/night	1.24	Inf	9.19	< 0.001
Day/dusk	0.81	Inf	− 7.08	< 0.001
Day/night	0.86	Inf	− 7.04	< 0.001
Dusk/night	1.06	Inf	2.15	< 0.001

The model equation is the following: Survival (Start, Stop, “Joining the aggregation”) ~ TEMPERATURE + FISH SIZE + TIME OF DAY + (1|Fish identity). The legend is the same as in Table 2

The probability of returning to the aggregation after an excursion significantly decreased with increasing temperature (Table 3c), especially when water temperature exceeded 9 °C (Fig. 6d). Therefore fish made longer excursions out of the aggregation when the temperature was high. The probability of coming back to the aggregation did not differ between fish of small and medium size (Table 3d) but significantly decreased for large fish (Table 3c, Fig. 6e). Large fish thus made longer excursions out of the aggregation than small or medium fish. Finally, the probability for fish of coming back into the aggregation decreased between dawn and day and between dusk and night (Table 3c, Fig. 6f). It increased between day and dusk and between night and dawn (Table 3c, Fig. 6f). Ranked in order of importance, fish thus went back into the aggregation first at dawn, then at dusk and last at night.

## Discussion

### Aggregation zone

Catfish aggregated across four winters for 1.5–2 months and showed a strong fidelity to the same zone representing 2–4% of the lake area. Even in other seasons, catfish have been shown to exhibit strong site fidelity [6, 10, 11, 52], but such an aggregation behavior has only been described in detail throughout a single year, and only once in a large river [4]. Site fidelity in fish can generally be linked to environmental features that provide a benefit to individuals, such as warmer temperatures [6, 10] or a refuge area for catfish [6], deep and slow pools for lake sturgeon *Acipenser fulvescens* [59], proximity to beds of emergent vegetation or open water formed by turbulence from a lake aerator for common carp *Cyprinus carpio* [46] or current updrafts that reduce energy expenditure for grey reef sharks *Carcharhinus amblyrhynchos* [44]. In our study however, the aggregation zone was not warmer than anywhere else in the lake and oxygen conditions were not limiting; moreover, a scan of the aggregation area with an acoustic camera (sonar 2D Oculus, 1.2 MHz [39]) on the 12th of February 2020 revealed no particular structure at the bottom of the lake. Finally, the main aggregation zone did not appear to be sheltered from the prevailing winds that blow from the north in this region and thus did not offer calmer water compared to the other parts of the lake.

### Aggregation dynamics

The formation and stability of the catfish aggregation in our study were closely linked to temperature. Similar observations have been made in common carp that aggregate when water temperature drops below 8 °C [29]. Fewer movements associated with the aggregation behavior is a way to save energy [19]. Aggregations were fairly stable across different times of day. Most movements took place at dusk and at night when some individuals left the aggregation and at dawn and at dusk when they came back. This is in agreement with the preferential nocturnal activity reported for catfish in the literature [6, 11]. The largest individuals spent more time outside of the aggregation. As the whole-organism metabolic rate of an individual increases with its body size (allometric equation, see [8]), large fish may need to leave the aggregation for feeding more often.

Even if not significant, the rank of arrival of individuals in the aggregation was more or less consistent across winters (*p* = 0.056), which could suggest that some individuals had a leading position (e.g. [28]). However, this was not the case for the rank of departure. On one hand, there was a tendency for some individuals to spend as much time as possible within the aggregation by arriving earlier and leaving later, and, on the other hand, there was a tendency for some others to minimize this time. Thus, leaders in the aggregation formation are not likely to be leaders in its dislocation, quite the contrary. The possible leading position was not linked to fish size contrary to what was observed for leadership in roach shoals [32] or for dominance in the catfish itself [53].

### Aggregation causes and consequences

Up to 100% of tagged fish gathered in the winter aggregation, which is much greater than the 23% reported for lake sturgeon [59] or the 70% reported for common carp [46]. Based on capture-recapture data (unpublished data), the estimated catfish population (individuals greater than 600 mm) in “Etang des Aulnes” is around 770 individuals (95% confidence interval [184; 1356]). As tagged individuals can be considered as a representative sample of the subadult and adult catfish population and as all tagged individuals at one point or another took part in the aggregation, a great proportion of the population could potentially gather there. This would thus make a huge winter aggregation of several hundreds individuals in comparison to the 44 individuals observed by Boulêtreau et al. [4]. In aggregations such as those that we observed, regrouping several hundreds individuals over 1.5–2 months, the level of competition between individuals could be considerably high [33]. This could be particularly true for catfish that have been reported to actively defend their access to resources in their core area [14, 52] and to be solitary foragers [11] during active periods. However, this competition could be here considerably dampened due to the cold season when catfish usually feed very little [14].

The cause or function of the aggregative behavior we observed remains unknown. The temperature was far below suitable temperatures for spawning (between 20 and 25 °C [56]), and the aggregation period occurs far too early to allow fish to identify potential mates for the spawning season that occurs several months later (usually in May–June in this lake). Unlike size-assortative schooling (e.g. [47]), individuals of various sizes could be found in the aggregation (range [727; 2150] mm in our tagged individuals), among which the smallest had probably not reached a refuge size against the largest individuals yet, since the prey-to-predator length ratio for catfish can reach 0.57 [66]. However, we do not know whether smaller individuals also aggregated. Some studies suggest that social interactions between conspecifics could play a role in fish aggregation, challenging the classical view of aggregation formation around floating devices [50]. Moreover, the site fidelity for aggregating could favor social interactions [73] like non-random aggregations in sharks [42]. As discussed above, regardless of their size, some individuals tended to stay longer in the aggregation while others seemed to shorten their stay. Irrespective of a possible hierarchy between individuals, longer stays in the aggregations could favor social interactions with conspecifics. These social interactions could regulate the stress between catfish, as was already shown in some species [1, 37]. However, Slavík and Horký [52] reported that catfish increase their energy consumption when in contact with conspecifics, assuming this was a stressful situation. This may not apply to our study though, since these authors made their observations on males only and in spring, when catfish return to normal activity and prefer to hunt solitarily [11]. Catfish have also been reported to decrease their activity in the presence of familiar conspecifics [53]. This raises the question of the role of animal personality and individual heterogeneity in collective behavior [30]. For example, shy individuals could be more likely to cooperate whereas bold ones could act more independently [13, 34]. Based on the calculation of a proximity index, Vanovac et al. [65] concluded that catfish do not display within-species interactions whereas common carp do, especially in winter. We may however question the sensitivity of this index to the sample size of fish. In fact, in Fig. 4 of their paper, which represents the location of species across seasons with kernel densities, one can clearly see a tendency for catfish to cluster in winter, similar to our observations.

Due to adults being at least twice as large as native fish predators, catfish are usually considered as a ‘giant’ top predator [14] and are suspected to threaten the fish communities [18, 23, 64]. Therefore, in numerous ecosystems, managers aim to control their population. However, a fundamental constraint of control methods is their lack of selectivity in specifically removing non-native fish species [7]. If our observations can be generalized to other locations and a considerable fraction of catfish populations also gather in the same zone during the coldest periods of the year, tagging only a few individuals could help locate and remove most individuals of a population (population control by the Judas technique) [3].

## Conclusion

Long-lasting winter aggregations of catfish, composed of individuals of various sizes and that likely concern a large proportion of the population, were shown to occur consistently every year at the same place in a restricted area of the lake. The aggregation formation and stability was closely linked to low temperatures. The area where the fish gathered was moderately deep and not different from other parts of the lake. Some individuals seemed to spend a longer time in the aggregation consistently every winter. Further studies are needed to explain this result, but one hypothesis is that it could correspond to different behavioral types, with certain individuals seeking sociality while others are more independent. In the end, this predictable seasonal grouping of individuals could provide an opportunity for lake managers to efficiently control catfish population if needed. As for species conservation, if the overwintering habitat is a critical requirement, it could constitute a bottleneck habitat, crucial to secure and maintain. The knowledge provided by our study has thus both academic and operational implications.

## Supplementary Information

**Additional file 1** Synchronized tracks of European catfish over the four winters 2017 to 2020 in “Etang des Aulnes” https://doi.org/10.57745/U7UG5D.

**Additional file 2** Time series of the mean distance between individuals in winters 2017 to 2020. The dates of structural changes over the 5-month time series (15 October–15 March) and their 95% confidence interval are labelled on the x-axis and represented by vertical dotted lines and interval at their basis (very narrow intervals are not visible).

**Additional file 3** Temperature differences between the main aggregation zone and the deepest point in the lake. Time series are represented over winters 2017 to 2020 (a–d). The solid black line represents the mean daily temperature differences at 0.5 m above the bottom between locations 38 and 42 (see Fig. 1) over the 5-month time series (15 October–15 March). Location 38 is inside the aggregation zone that showed off each winter. Location 42 corresponds to the deepest point in the lake and stands as a reference point. The dates of aggregation given by the breakpoint detection algorithm are represented by vertical blue dotted lines. The period extended to the formation and dislocation of the aggregation is delimited by vertical blue solid lines.

**Additional file 4** Temperature differences between the secondary aggregation zone and the deepest point in the lake. Time series are represented over winter 2019. The solid black line represents the mean daily temperature differences at 0.5 m above the bottom between locations 5 and 42 (see Fig. 1) over the 5-month time series (15 October–15 March). Location 5 is very close to the secondary aggregation zone that showed off only in winter 2019. Location 42 corresponds to the deepest point in the lake and stands as a reference point. The dates of aggregation given by the breakpoint detection algorithm are represented by vertical blue dotted lines. The period extended to the formation and dislocation of the aggregation is delimited by vertical blue solid lines.

## Data Availability

The datasets analyzed during the current study are available from the corresponding author on reasonable request.
