# Supplementary material for: Overwintering aggregation patterns of European catfish Silurus glanis
Source: Mov Ecol. 2023 Feb 7;11:9. doi: 10.1186/s40462-023-00373-6 (PMC9903427; doi:10.1186/s40462-023-00373-6)
Supplement: Supplementary file 1 — Additional file 1 Synchronized tracks of European catfish over the four winters 2017 to 2020 in “Etang des Aulnes” https://doi.org/10.57745/U7UG5D. [file 40462_2023_373_MOESM1_ESM.pdf]

**Supplementary material 1** Synchronized tracks of European catfish over the four winters 2017-2020 in "Etang des Aulnes"

https://doi.org/10.57745/U7UG5D
