# Supplementary material for: Overwintering aggregation patterns of European catfish Silurus glanis
Source: Mov Ecol. 2023 Feb 7;11:9. doi: 10.1186/s40462-023-00373-6 (PMC9903427; doi:10.1186/s40462-023-00373-6)
Supplement: Supplementary file 2 — Additional file 2 Time series of the mean distance between individuals in winters 2017 to 2020. The dates of structural changes over the 5-month time series (15 October–15 March) and their 95% confidence interval are labelled on the x-axis and represented by vertical dotted lines and interval at their basis (very narrow intervals are not visible). [file 40462_2023_373_MOESM2_ESM.pdf]

Supplementary material 2 Time series of the mean distance between individuals in winters 2017 to 2020.

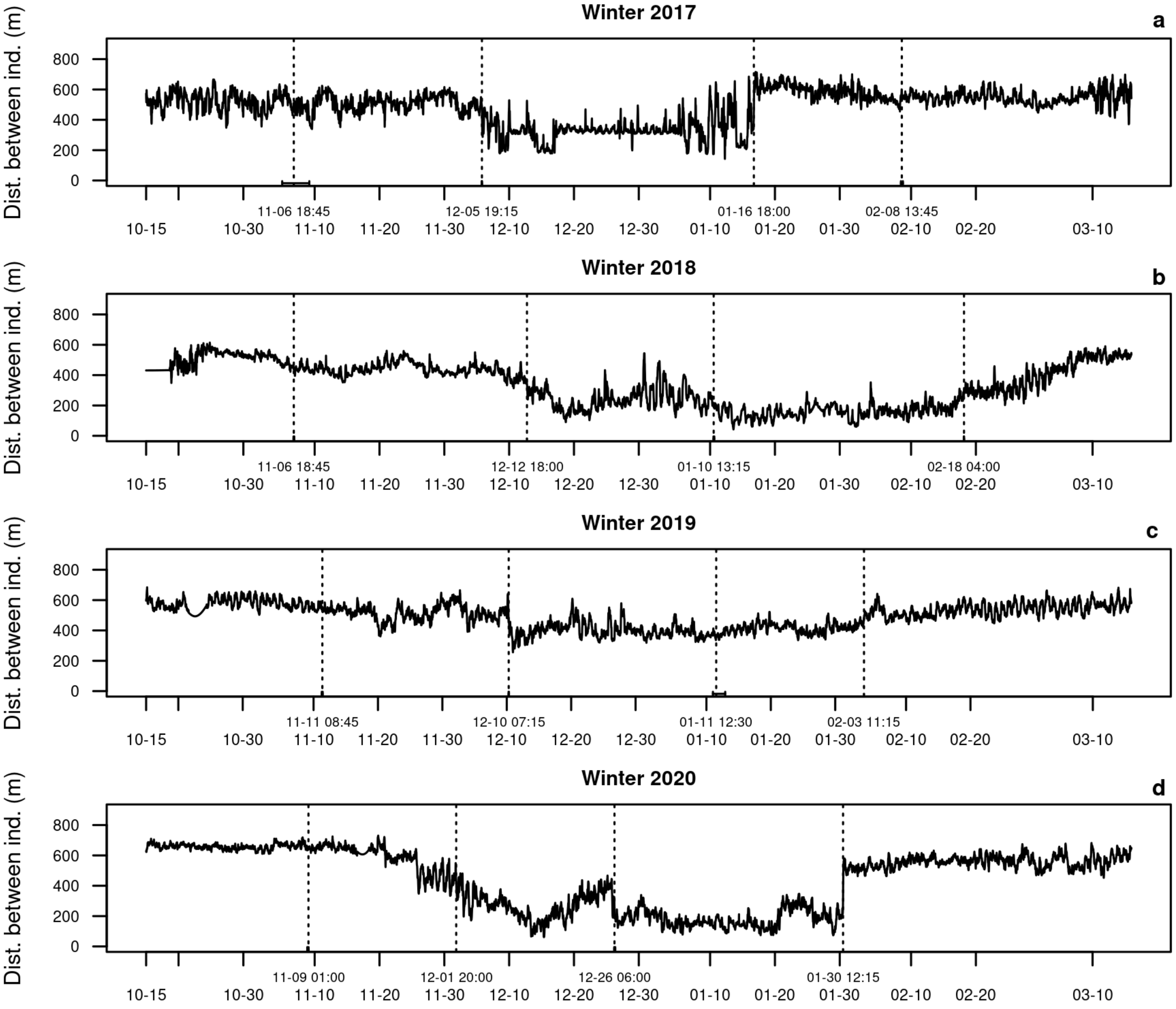

The dates of structural changes over the 5-month time series (15 October-15 March) and their 95% confidence interval are labelled on the x-axis and represented by vertical dotted lines and interval at their basis (very narrow intervals are not visible).
