# Supplementary material for: Overwintering aggregation patterns of European catfish Silurus glanis
Source: Mov Ecol. 2023 Feb 7;11:9. doi: 10.1186/s40462-023-00373-6 (PMC9903427; doi:10.1186/s40462-023-00373-6)
Supplement: Supplementary file 3 — Additional file 3 Temperature differences between the main aggregation zone and the deepest point in the lake. Time series are represented over winters 2017 to 2020 (a–d). The solid black line represents the mean daily temperature differences at 0.5 m above the bottom between locations 38 and 42 (see Fig. 1) over the 5-month time series (15 October–15 March). Location 38 is inside the aggregation zone that showed off each winter. Location 42 corresponds to the deepest point in the lake and stands as a reference point. The dates of aggregation given by the breakpoint detection algorithm are represented by vertical blue dotted lines. The period extended to the formation and dislocation of the aggregation is delimited by vertical blue solid lines. [file 40462_2023_373_MOESM3_ESM.pdf]

Supplementary material 3 Temperature differences between the main aggregation zone and the deepest point in the lake.

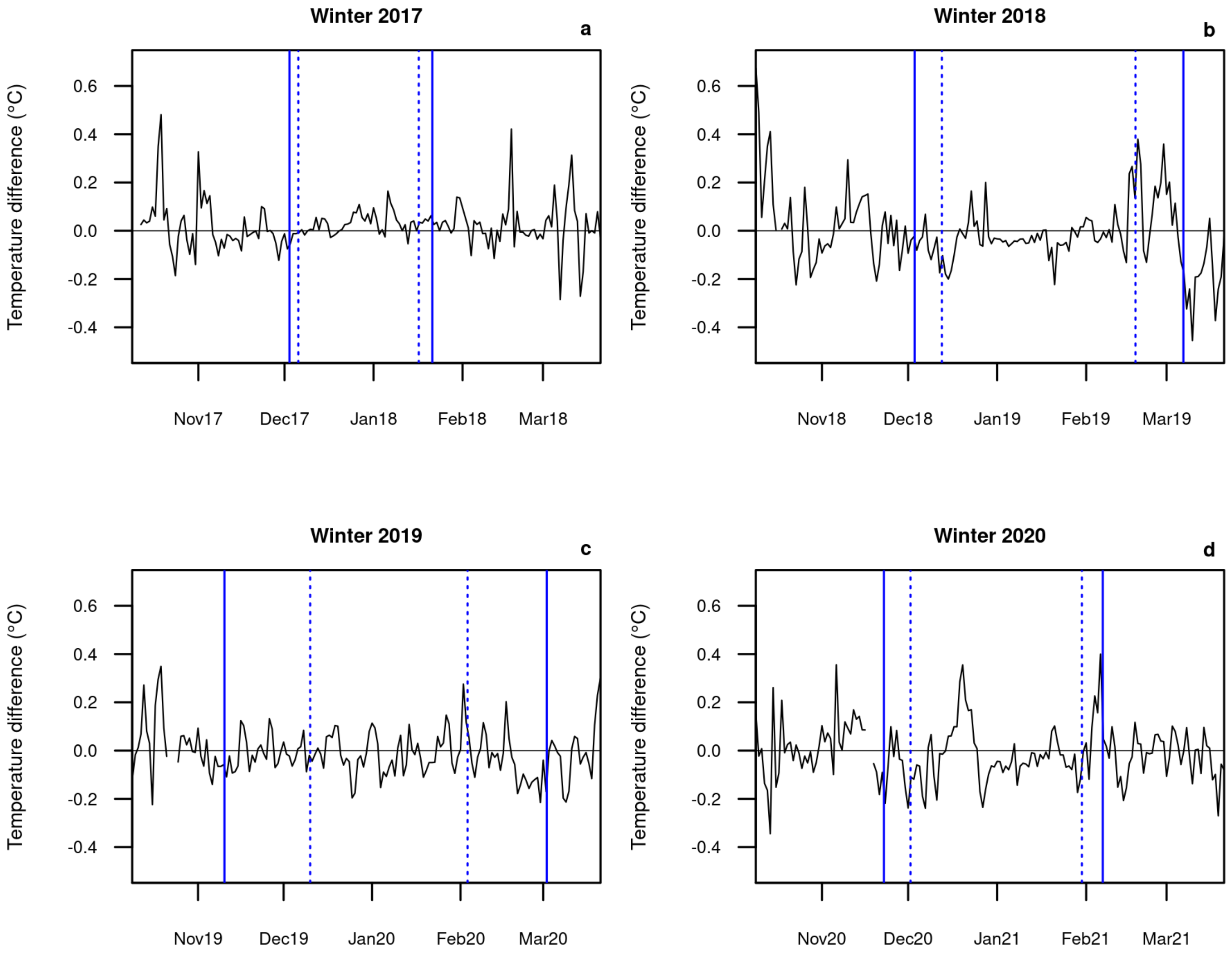

Time series are represented over winters 2017 to 2020 (a–d). The solid black line represents the mean daily temperature differences at 0.5 m above the bottom between locations 38 and 42 (see Figure 1) over the 5-month time series (15 October-15 March). Location 38 is inside the aggregation zone that showed off each winter. Location 42 corresponds to the deepest point in the lake and stands as a reference point. The dates of aggregation given by the breakpoint detection algorithm are represented by vertical blue dotted lines. The period extended to the formation and dislocation of the aggregation is delimited by vertical blue solid lines.
