# Supplementary material for: Overwintering aggregation patterns of European catfish Silurus glanis
Source: Mov Ecol. 2023 Feb 7;11:9. doi: 10.1186/s40462-023-00373-6 (PMC9903427; doi:10.1186/s40462-023-00373-6)
Supplement: Supplementary file 4 — Additional file 4 Temperature differences between the secondary aggregation zone and the deepest point in the lake. Time series are represented over winter 2019. The solid black line represents the mean daily temperature differences at 0.5 m above the bottom between locations 5 and 42 (see Fig. 1) over the 5-month time series (15 October–15 March). Location 5 is very close to the secondary aggregation zone that showed off only in winter 2019. Location 42 corresponds to the deepest point in the lake and stands as a reference point. The dates of aggregation given by the breakpoint detection algorithm are represented by vertical blue dotted lines. The period extended to the formation and dislocation of the aggregation is delimited by vertical blue solid lines. [file 40462_2023_373_MOESM4_ESM.pdf]

Supplementary material 4 Temperature differences between the secondary aggregation zone and the deepest point in the lake.

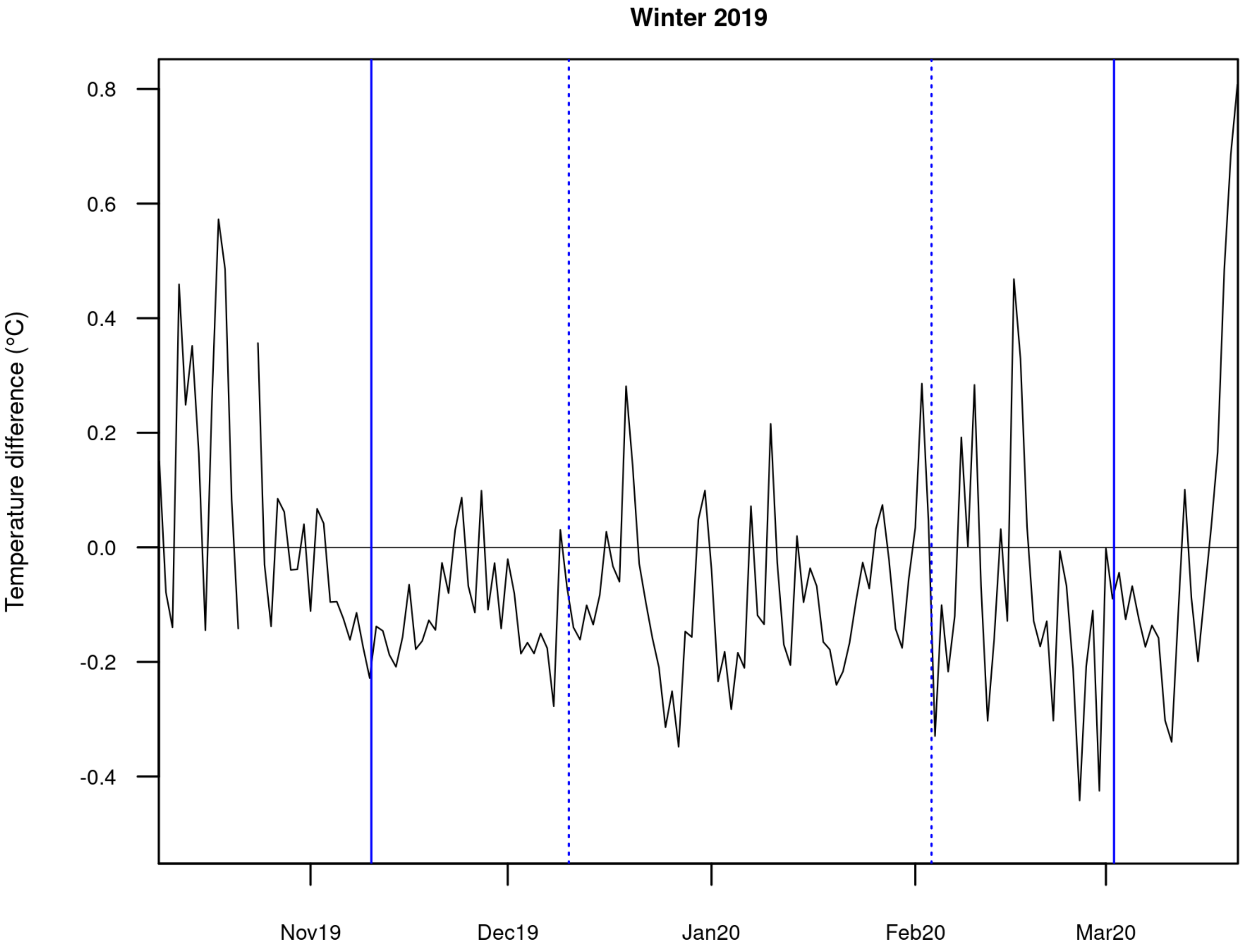

Time series are represented over winter 2019. The solid black line represents the mean daily temperature differences at 0.5 m above the bottom between locations 5 and 42 (see Figure 1) over the 5-month time series (15 October-15 March). Location 5 is very close to the secondary aggregation zone that showed off only in winter 2019. Location 42 corresponds to the deepest point in the lake and stands as a reference point. The dates of aggregation given by the breakpoint detection algorithm are represented by vertical blue dotted lines. The period extended to the formation and dislocation of the aggregation is delimited by vertical blue solid lines.
